# Computational Insight on the Interaction of Common Blood Proteins with Gold Nanoparticles

**DOI:** 10.3390/ijms22168722

**Published:** 2021-08-13

**Authors:** Francesco Tavanti, Maria Cristina Menziani

**Affiliations:** 1 CNR-NANO Research Center S3, Via Campi 213/a, 41125 Modena, Italy; 2 Department of Chemical and Geological Sciences, University of Modena and Reggio Emilia, Via Campi 103, 41125 Modena, Italy; mariacristina.menziani@unimore.it

**Keywords:** nanoparticle, hemoglobin, albumin, protein-corona, computer simulation, molecular dynamics, coarse-grained model

## Abstract

Protein interactions with engineered gold nanoparticles (AuNPs) and the consequent formation of the protein corona are very relevant and poorly understood biological phenomena. The nanoparticle coverage affects protein binding modalities, and the adsorbed protein sites influence interactions with other macromolecules and cells. Here, we studied four common blood proteins, i.e., hemoglobin, serum albumin, α1-antiproteinase, and complement C3, interacting with AuNPs covered by hydrophobic 11-mercapto-1-undecanesulfonate (MUS). We use Molecular Dynamics and the Martini coarse−grained model to gain quantitative insight into the kinetics of the interaction, the physico-chemical characteristics of the binding site, and the nanoparticle adsorption capacity. Results show that proteins bind to MUS−capped AuNPs through strong hydrophobic interactions and that they adapt to the AuNP surfaces to maximize the contact surface, but no dramatic change in the secondary structure of the proteins is observed. We suggest a new method to calculate the maximum adsorption capacity of capped AuNPs based on the effective surface covered by each protein, which better represents the realistic behavior of these systems.

## 1. Introduction

Engineered nanoparticles (NPs) gained the attention of several branches of science due to their unique physical, chemical, and electrical properties, and they have been used in several emerging applications, such as biomedicine and catalysis [1,2]. According to Wei and Yan, in 2016, there were more than 3′000 nanomaterial-based products on the market available to consumers for use in healthcare, fitness, automotive, electronics, and food, and their number was growing rapidly [3]. The use of all of these products will increase the risk of exposure to these NPs and to the side effects that they can have on the human body because they can be inhaled or enter in the body through the food. The understanding of the interactions of NPs with the biological medium will help to design newer and safer nanomaterials with reduced toxicity and to develop nanomedicine applications such as drug delivery to well-defined biological sites [2,4]. The main problem in developing non-toxic and effective nanomaterials is caused by the lack of knowledge regarding nanoparticle interactions with the biological medium. It is well-known that when a NP comes in contact with a physiological environment such as blood, proteins and macromolecules readily interact and adsorb over the NP surface, creating a layer of biomolecules called the “corona” [5,6]. The protein corona consists of several proteins. Each protein exhibits a different affinity, binding site, and population depending on the NP features, on its own relative abundance, and on the biological environment [5,6]. The protein corona mediates the interactions with cells and with other biological entities that may come into contact with this new bio-aggregate [6,7,8]. Moreover, the NP size, shape, and its capping layer of ligands (if present) drive the interactions with proteins, making this system very difficult to understand in all aspects due to the several factors that are involved. For example, citrate capped-gold NPs (AuNP) mainly interact through electrostatic interactions that drive the adsorption of proteins and their binding site [9,10,11,12,13,14,15]. On the contrary, it has been shown that some common blood proteins, such as serum albumin and hemoglobin, have a high affinity to hydrophobic AuNPs, and there are several studies that prove that mixed hydrophobic/hydrophilic AuNPs can pass through the cellular membrane [16,17]. Recently, Cox et al. [18] studied the evolution of the protein corona around AuNPs capped with 11-mercapto-1-undecanesulfonate (MUS) that confers a strong hydrophobic nature to the AuNP before and after Brain–Blood Barrier (BBB) passage. They showed that the corona protein composition dramatically changes with a high probability of finding serum albumin after passage across the BBB. The same kind of MUS-capped AuNPs have been employed as virucidal agents for the herpes simplex virus, human papilloma virus, respiratory syncytial virus, dengue [19], and recently, for the SARS-CoV2 virus [20] without toxic side effects, suggesting that they can be good candidates for innovative and safe nanomedicine applications. However, the interactions of common blood proteins with MUS-capped AuNPs are not well understood, but they are of fundamental importance to progress in this field. In this work, we elucidated the interactions of common blood proteins with all-MUS AuNPs through the use of computer simulations. Human serum albumin, hemoglobin, complement C3, and α1-antiproteinase have been chosen as test cases due to their high abundance in the blood stream and their different affinity with MUS-capped AuNPs [19,21]. The results show that hydrophobic interactions play the most relevant role in the AuNP–protein binding, and we then propose a new methodology to evaluate the maximum adsorption capacity of NPs by means of classical Molecular Dynamics (MD) simulations.

## 2. Results

### 2.1. Interaction Sites of Proteins with AuNP

The spontaneous binding of proteins to the AuNP was observed for all types of proteins during the simulations, which were conducted without any bias, i.e., no external driving force was applied to proteins to bind to the AuNP. As previously found, MUS ligands interact each other due to their hydrophobic nature, and they aggregate each other [22]. Proteins interact with the side of the MUS aggregates and with the free space over the AuNP surfaces. The binding site for each protein was identified by only considering amino acids whose backbone beads have a distance less than 4.5 Å from the all-MUS AuNP, and these computed on the last frame of the simulation when the binding was stable. Most of the contact of the proteins with the all-MUS AuNP were achieved by hydrophobic amino acids, while the percentage of positively and negatively charged amino acids was quite low and depended on the binding site of the protein. The results summarized in Table 1 show that there are significant differences between the four proteins. This is not only due to the different number of each amino acid for each protein but principally on the distribution of amino acids on the surface of the protein and on how they form hydrophobic and hydrophilic regions. This trend is completely different from that observed in previous works, which employed citrate-capped AuNP [9,10,11,12,13,23]. In these cases, electrostatic interactions established by charged amino acids, such as Lysine, are the driving forces for protein binding due to the presence of a strongly charged citrate layer on the AuNP. At the bottom of Table 1, the most probable binding sites for each protein and the average value of the Potential of Mean Force (PMF) obtained through the umbrella sampling technique are reported. By taking the binding strength for each protein averaged over the possible binding sites, we observed that complement C3 is the protein with the highest values of the PMF due to its wide binding site with the AuNP. Hemoglobin, serum albumin, and α1-antiproteinase have similar PMF values despite having a different number of atoms and different secondary structures. This means that the binding strength is independent from the protein size and secondary structure, but it does dependent on the local shape and the physico-chemical characteristics of the protein.

#### 2.1.1. Antiproteinase

**Figure 1:**
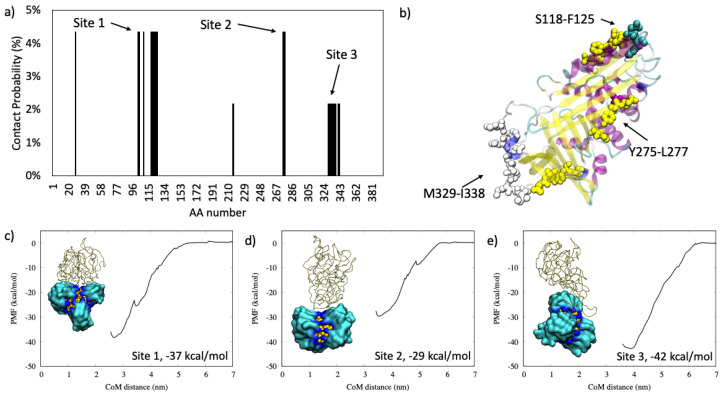
In panel (**a**): the contact probability for each residue upon α1-antiproteinase binding to all-MUS AuNP. In panel (**b**): the graphical representation of the most probable binding sites in VdW, colored according to the protein secondary structure. In panels (**c**–**e**): the three binding modalities with the relative PMF curve.

We observed that for α1-antiproteinase, there are three well defined binding sites located at S118-F125 (called Site 1), Y275-L277 (called Site 2), and at M329-I338 (Site 3), as shown in Figure 1. From the representative poses in Figure 1c–e, it is clear that the binding at Site 2, where the protein lies perpendicular to the AuNP surface, is less strong than at Site 1, which is due to the small number of interacting residues.

#### 2.1.2. Serum Albumin

**Figure 2:**
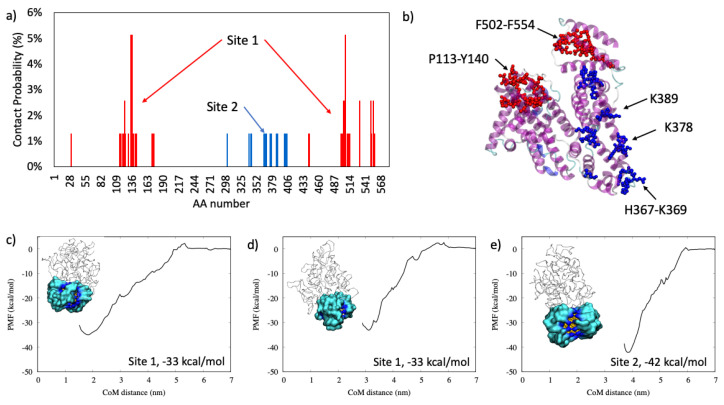
In panel (**a**): the contact probability for each residue upon serum albumin binding to all-MUS AuNP. Amino acids belonging to Site 1 are colored in red, while those of Site 2 are colored in blue. In panel (**b**): the graphical representation of the most probable binding sites in VdW, colored according to the binding site. In panels (**c**–**e**): the two binding modalities with the relative PMF curve.

An opposite trend with respect to the binding of α1-antiproteinase is observed for serum albumin, as shown in Figure 2. The Site 1 of the binding is very broad, spanning from P113-Y140 to F505-F554, and the average PMF is −36 kcal/mol. For binding Site 2, the PMF is stronger (−42 kcal/mol) although the binding site is composed of fewer amino acids: from H367 to K369, K378 and K389.

#### 2.1.3. Complement C3

**Figure 3:**
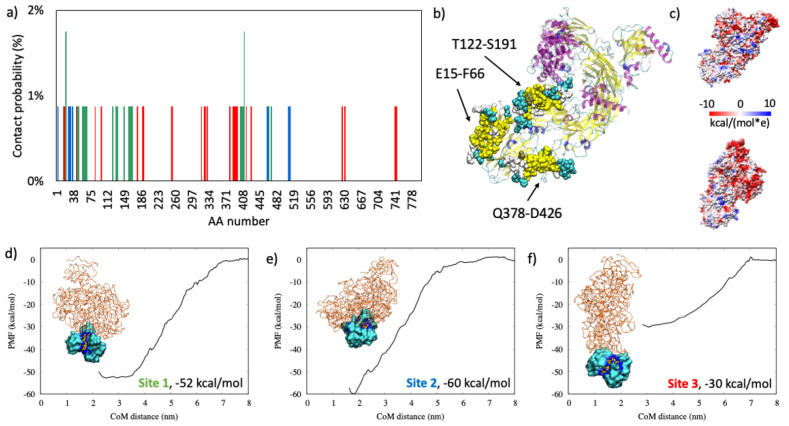
In panel (**a**): the contact probability for each residue upon complement c3 binding to all-MUS AuNP. Each color represents a different binding site. Amino acids over 800 are removed for clarity because their contact probability was zero. In panel (**b**): the graphical representation of the most probable binding sites in VdW, colored accordingly to the protein secondary structure. In panel (**c**): the coulombic surface of complement C3 as viewed from the front (top) and back (bottom). Colors represent the coulombic potential as shown in the legend. In panels (**d**–**f**): the three binding modalities with the relative PMF curve.

Complement C3 mainly interacted with the all-MUS AuNP through the residues going from E15 to D426, and we obtained three different binding sites. These three regions are close each other, but they belong to different binding modalities that orient the protein in three different ways as, shown in Figure 3, and that give different binding strength. The region on the C-terminal was never found to interact with the AuNP. From the calculation of the Coulombic surfaces reported in Figure 3c, we observed that this region has a strong propensity to be negatively charged, while the binding regions show neutral behavior. It is worth noting that binding Site 3 is very extended but has a low PMF value. Conversely, binding Site 2 is restricted to few amino acids but shows the highest binding strength (−60 kcal/mol), which is also the case if it is compared to other proteins.

#### 2.1.4. Hemoglobin

**Figure 4:**
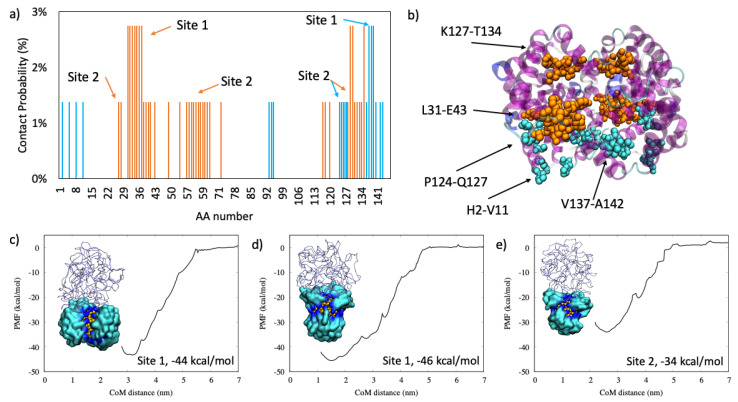
In panel (**a**): the contact probability for each residue upon hemoglobin binding to all-MUS AuNP. In panel (**b**): the graphical representation of the most probable binding sites in VdW, colored according to the different hemoglobin chains: chain A is in orange, and chain b is in cyan. In panels (**c**–**e**): the two binding modalities with the relative PMF curve.

Hemoglobin interacts with two main and extended binding sites, reported in Figure 4. The main binding site is located on residues L31-E43 and V137-A142, and it has a higher PMF with respect to the Site 2. In this case, the binding site is mainly located on residues K127-T134, and the protein is oriented upward with respect to the AuNP surface, as shown in Figure 4e. Hemoglobin interacts with almost all of the amino acids with the AuNP because this protein is small and globular, and the same trend was observed for hemoglobin interacting with citrate-capped AuNP [24].

**Figure 5:**
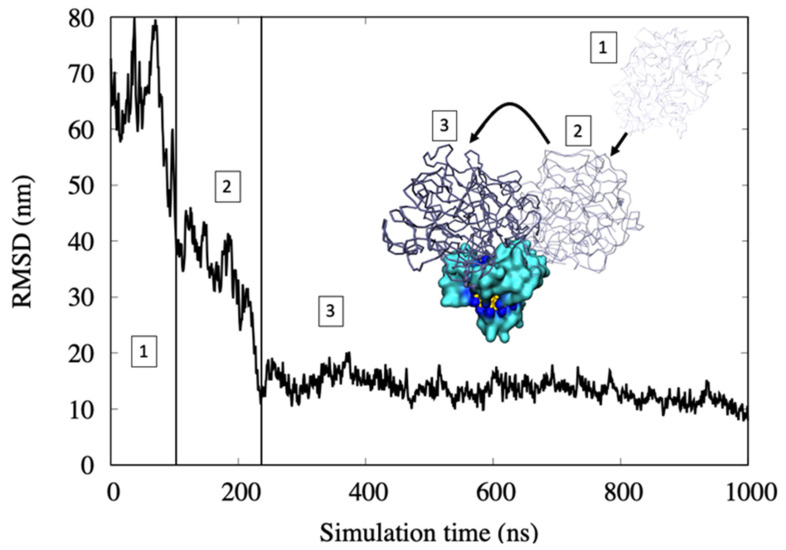
The Root Mean Square Deviation (RMSD) of the hemoglobin showing the three-steps of binding to AuNP. In step 1, the protein is far away from the AuNP. In step 2, the protein makes its first contact with the AuNP over the heads of the ligands. In step 3, the protein finds the most stable binding site.

Moreover, in the case of hemoglobin, we observed that the adsorption over all-MUS AuNP happens in three steps, as previously found for citrate-capped AuNP [9,25]. Figure 5 shows the RMSD of the center of mass of the hemoglobin during the adsorption on the AuNP and where the binding happens through the following process:

1. In the first step, protein motion in water is regulated by a diffusion regime. Here, the RMSD values are high due to the high mobility of the hemoglobin.
2. The first binding with the all-MUS AuNP is with the MUS ligands that cap the surface. The RMSD goes down fast due to the lower mobility of the protein. This binding is weak because only a few amino acids interact with the ligand chains.
3. The protein rearranges its position over the AuNP and, by being involved in binding several chains of MUS ligands, yields strong hydrophobic interactions. Here, the RMSD reaches a lower and more stable value, indicating a stable binding with the AuNP.

### 2.2. Conformational Changes

After binding, proteins do not show significant changes in their secondary structure but do minor rearrangements due to the interaction with the AuNP. The gyration radius, R_gyra_, listed in Table 2, is a measure of the protein compactness and sphericity. By comparing R_gyra_ before and after the binding of the four proteins studied with the AuNP, we can observe that it is only the serum albumin that becomes slightly less compact upon binding, while small effects are observed for the other three proteins.

**Figure 6:**
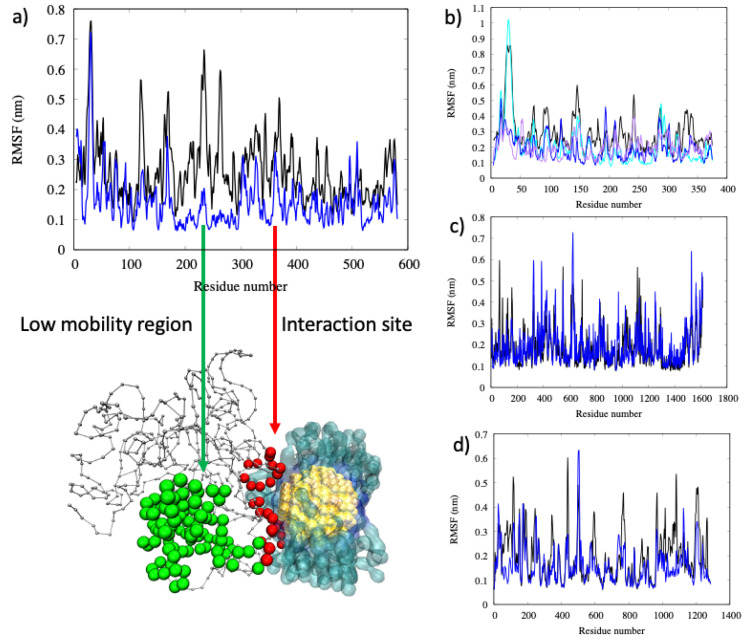
Root mean square fluctuation (RMSF) of (**a**) serum albumin before (in black) and after (in blue) binding with the all-MUS AuNP. At the bottom the graphical representation of the interaction site (spheres in red) and the region with low mobility (spheres in green) is shown. the protein backbone is represented in gray, and the MUS ligands are represented as a transparent blue surface to facilitate the observation of the protein binding region. Panels (**b**–**d**) reports the RMSF for α1-antiproteinase, complement C3, and hemoglobin, respectively. panel b) reports the three replicas of the simulation in blue, cyan, and purple, while for albumin, complement C3, and hemoglobin only one replica is shown for clarity.

The root mean square fluctuation (RMSF) of the protein reported in Figure 6 is a measure of the mobility of each amino acid. By comparing the RMSF before and after binding, we observed that small proteins, such as α1-antiproteinase, are less flexible after binding, in particular, as expected, this can be observed for residues in contact with the AuNP. In the case of medium-size proteins such as serum albumin, the amino acids close to the AuNP (residues from 350 to 400) have reduced mobility with respect to the free protein. Interestingly, the amino acid regions comprised from residues 200 to 300 show a significantly lower mobility despite it lying farther from the binding site (this region is highlighted with green spheres in Figure 6), while all of the remaining amino acids remain flexible, as is the case in the free protein. This finding suggests that binding to AuNP could alter the protein structure directly involved and in near proximity to some extent, but long-range effects cannot be neglected. Moreover, for bigger proteins, as is the case with complement C3, we observed very small changes in the RMSF in close proximity to the binding site and to the most flexible residues, such as those forming loops that lie in the exterior of the protein, as shown in Figure 6c.

### 2.3. Maximum AuNP Adsorption Capacity

**Figure 7:**
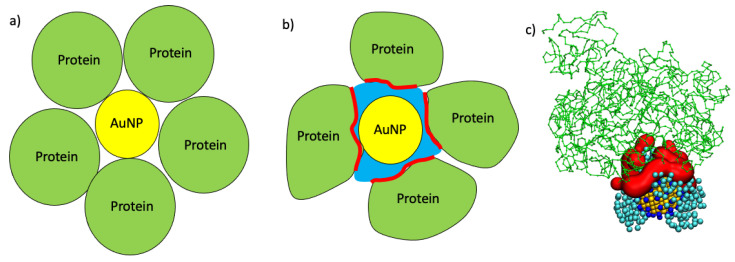
In panel (**a**), a sketch of the maximum number of proteins that can absorb on the AuNP in the geometrical case where both the AuNP and the proteins are treated as rigid spheres is shown. In panel (**b**), a sketch of a real case where the AuNP is surrounded by the ligands (in blue) and where the proteins are not perfect spheres but instead adapt to the AuNP surface is shown. The red line represents the binding site of the proteins. Panel (**c**) shows the adsorption of complement c3 (in green) over the all-MUS AuNP, where the interacting surface is highlighted in red.

The number of proteins that can form the protein corona around nanoparticles can be obtained from the equations proposed by Wang et al. [25], Dell’Orco et al. [26], and Calzolai et al. [10], which showed good agreement with experiments and simulation results [9,11,24]. However, these approaches are based on geometrical considerations, for example, by considering spherical proteins as rigid spheres, and they do not take into account possible conformational changes and the effect of non-spherical proteins, such as in the case of complement C3, see Figure 7a. In order to considering non-spherical proteins besides protein conformational changes upon binding to AuNP, we developed a new formulation for the maximum number of proteins that can absorb over an AuNP, see sketch in Figure 7b. For each protein we computed the Solvent Accessible Surface Area (SASA) before ($SASA_{initial}$) and after ($SASA_{final}$) AuNP binding. The surface occupied by a single protein on the AuNP surface (∆SASA) is given by Equation (1):

(1) $$∆SASA=SASA_{initial}-SASA_{final}$$

The maximum number of proteins (*N_max_*) is the ratio between the $SASA_{ligands-NP}$ and ∆SASA:

(2) $$N_{max}=\frac{SASA_{ligands-NP}}{∆SASA}$$

where $SASA_{ligands-NP}$ is the SASA of the complex AuNP covered with all-MUS ligands.

This Equation (2) is based on the effective surface occupied by a single protein over the AuNP obtained by MD simulations, and it is intrinsically dependent on not only the protein shape and size but also on the affinity of the protein binding site to AuNP and on the possible displacement of the ligands that cap the AuNP. The displacement of the ligands and the adaptation of the proteins to the NP surface have already been found for not only the proteins adsorbed on NPs but also for those on flat surfaces due to the interactions with the metals and with the ligands that cap the nanomaterial [9,12,13,23,27,28,29]. As shown in Figure 7c, proteins as Complement C3 tend to interact with capped AuNPs in the site that yields more efficient binding with the effect of increasing the contact surface.

In this study, the AuNP has a core diameter of 2.2 nm that is increased to 4.3 nm if the all-MUS capping agent is considered, which is in the same order of magnitude as the size of the proteins. Table 3 lists the size of each protein and the N_max_ computed according to previous works of Wang et al. [25], Calzolai et al. [10], and Dell’Orco et al. [26] and by the proposed method reported in Equation (2). We can observe that the methods of Calzolai and Dell’Orco [10,26] give similar values, while the method of Wang [25] is the one with lower values of N_max_. Our study shows that the maximum adsorption capacity does not depend on the mass of the protein nor on its size or compactness but on its ability to adapt to the AuNP surface. In fact, as shown in Figure 1, Figure 2, Figure 3 and Figure 4, we observed similar values for hemoglobin and serum albumin that have a similar surface fingerprint on the binding site despite having a very different size, while this is not true for α1-antiproteinase and complement C3.

## 3. Discussion

The binding of proteins with AuNPs is strongly dependent on the ligands that cap the metal surface. The understanding of how ligand capped-AuNPs interact with different kind of proteins is of fundamental importance in the design of new and more effective nanotechnologies for an efficient drug delivery methodology. We employed the Martini Coarse Grained model to describe the interactions of all-MUS AuNPs with four different common blood proteins: α1-antiproteinase, serum albumin, hemoglobin, and complement C3. A AuNP was covered with MUS ligands that are strongly hydrophobic and they conferred a hydrophobic surface to the AuNP, as previously found by Chew et al. [22] for different kinds of capping agents. We observed that hydrophobic interactions play a major role due to the interactions with hydrophobic MUS ligands, while charged amino acids show small contact probabilities. This trend is completely different from what was previously found for citrate-capped AuNPs, where electrostatic plays the most relevant role in the binding process and for AgNPs, where interactions with albumin are mainly given by the Coulombic term [30]. This difference is due to the different NP coverage that can greatly affect binding with proteins. In a recent work, Yu et al. [21] studied how different AuNP capping ligands can determine the kind of adsorbed proteins using TEM, SDS page, and Nano-LC-MS/MS measurements, finding that the more hydrophobic the NP, the more proteins that can bind to it. Moreover, they showed that hemoglobin and serum albumin strongly interact with hydrophobic AuNPs, while complement C3 seems to prefer AuNP with a medium hydrophobicity.

We observed that these four proteins do not undergo to significant conformational changes in their secondary and tertiary structures, with a only a small tendency for α1-antiproteinase to become more compact after the binding, due to interactions with the MUS ligands over the AuNP surface. This finding is in agreement with previous experimental and computational studies on the interaction of albumin and hemoglobin with capped AuNPs where the conformational changes are related to the size of the AuNP [24,31]. A recent mixed experimental and computational work showed that serum albumin has no or very small conformational changes after binding over 4.5nm diameter AgNPs [30]. In this work, we only observed minor readjustments of the amino acids side chains that tend to lie over the MUS surface, as described by small changes of the gyration radius. Interestingly, we observed that serum albumin is less flexible after the binding in the region from amino acids 200 to 300, which is quite far from the binding region. This could be an indirect effect of the loss of flexibility in the structure in contact with the AuNPs that affect the degree of freedom of this region, which is very flexible for free proteins. This finding suggests that the interactions of proteins with NPs not only affect the binding site but also affect the region of the protein that are not responsible of the binding and that are exposed to the solvent mediating the interactions with other biological macromolecules or cells. For example, the binding site of complement C3 with the AuNP leaves the binding site with the staphylococcal complement inhibitor free [32]. For this reason, is very important to understand how proteins interact with NPs and how this binding affects the characteristics of other portions of the protein surface in order to lower side effects when they are used in new applications [7].

All proteins show a strong binding (from −30 kcal/mol to −60 kcal/mol) that depends on both the number of amino acids at the interface with the all-MUS AuNP and on their hydrophobicity. Our findings suggest that complement C3 is the one with the highest binding strength, while the other three proteins have similar values. By taking the number of atoms for each protein into account, we can observe that α1-antiproteinase, despite the lower mass and lower number of atoms, has a PMF value similar to serum albumin that is almost the double in size, confirming the hypothesis that it is not the size of the protein that drives the binding strength with AuNPs, but mainly the affinity of each protein to that specific AuNP.

Hemoglobin, serum albumin, and α1-antiproteinase are all quite compact proteins and have similar gyration radius. These three proteins adapt to the MUS ligands during binding, finding the best pose over the AuNP surface. Conversely, complement C3 is an extended protein with a higher gyration radius, and it tends to wrap around the AuNP during the interaction. This finding is also supported by both the PMF that is higher for complement C3 due to the higher number of amino acids involved in the interaction and by the maximum number of proteins that can bind to the AuNP that reflect the higher surface covered by this protein.

The well-known three steps model for adsorption of proteins over NPs [9,25] were also observed in this study showing, that proteins tend to maximize hydrophobic interactions.

Finally, we described a new method to estimate the maximum number of proteins that can absorb over a given AuNP. This methodology relies on the effective surface occupied by the given protein over the effective AuNP surface, considering not only the possible conformational changes of proteins, but also the shape of the AuNP and of the ligands that occupy its surface. This method overcame some limitations of previous methodologies based only on geometrical considerations and that do not take the softness of both proteins and ligands layer over AuNP into account. Results from our simulations show that the values that were obtained are in between the ones obtained by method of Wang et al. [25] and by those from Calzolai et al. [10] and Dell’Orco et al. [26]. We observed that up to three complement C3 can be absorbed due to the small interaction site with respect to the size of the AuNP, while up five albumin and six α1-antiproteinase could bind. In these last cases, the proteins tended to adapt to the AuNP surface covering a significant amount of the NP surface, so both the proteins and the capped AuNPs could not be treated as geometrical rigid spherical bodies. The case of complement C3 is peculiar. The method of Wang et al. [25] predicts that only one protein can bind, while the other two methods [10,26] predict a number between 7 and 10. In the first case was clear that the protein could not cover the entire AuNP surface unless it underwent important conformational changes so that at least two protein could adsorb. The other methods produce a high number of proteins, but these predict a very compact binding of all of the proteins that is not possible for complement C3 due to its size and extended shape. However, we previously found that these methods are in a better agreement with the computer simulation of the formation of the protein corona when employing bigger NPs covered with citrates that poorly affect their spherical shape [9,11,24]. In fact, citrates are of small size, and they lie over the NP surface, not modifying the spherical shape of the NP [13].

## 4. Materials and Methods

**Figure 8:**
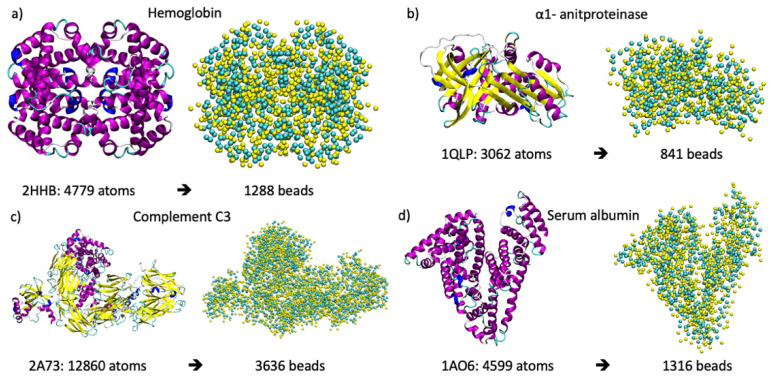
In each panel on the left, the representations of the three-dimensional structures of the proteins where secondary structure elements are represented in cartoons are shown, and on the right, the CG Martini model is shown, where cyan spheres are related to the backbone beads and the yellow is related to the side chains. In panel (**a**) hemoglobin; (**b**) α1-antiproteinase; (**c**) serum albumin; and (**d**) complement C3. Below each protein, the PDB ID, the number of atoms at the atomistic level, and the number of beads at the cg level are shown.

In order to understand the interactions of common human blood plasma proteins with monolayer-capped AuNPs, we employed classical MD simulations. The atomistic structures of the proteins were retrieved from the PDB database [33], and they are reported in Figure 8 with their relative number of atoms and number of CG Martini beads. Hemoglobin is a globular protein involved in the oxygen transport chain formed by interconnected α-helices [34]. α1-antiproteinase (or α1-antitrypsin) can inhibit different proteins, such as enzymes, by covalently binding to them and its secondary structure, which is composed of both α-helices and β-sheets [35]. Serum albumin is a protein that delivers fatty acids in the blood stream, and its tertiary structure is characterized by long and interconnected α-helices [36]. Complement C3 belongs to the α2-macroglobulin family and interacts with a large number of both complement ad non-complement proteins. It is a 15 nm long protein that is characterized by 11 β-sheet domains and by a short barrel of α-helices [37]. All of the protein structures, their relative secondary and tertiary structure organization, and the CG model are reported in Figure 8.

**Figure 9:**
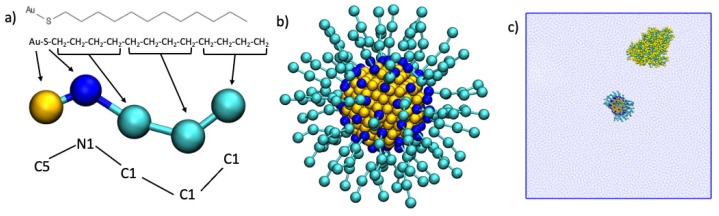
In panel (**a**), the atomistic representation and the cg Martini model mapping of the MUS ligand is shown. in panel (**b**), the cg representation of the all-MUS AuNP before running the simulation is shown. In panel (**c**), the system with the AuNP fixed in the center of the simulation box after some 1s of simulation is shown, where the protein is represented by yellow and cyan beads, and the Martini water is represented with small blue points.

The gold nanoparticle was built as a truncated octahedron with a face-centered cubic (fcc) lattice with a core diameter of 2.2nm and consisting of 314 atoms [17]. The gold core is capped at the atomistic level with MUS ligands, as reported in Figure 9. To obtain the CG model, we mapped the atomistic NP according to the Martini scheme [38], according to the work of Salassi et al. [17]. In particular, both the gold and sulfur atoms of the MUS are mapped at a ratio of 1:1, and they were kept fixed during the simulations. The MUS ligands were mapped at a ratio of 4:1 by employing three “C1” beads to represent the carbon chain and “N0” for the S atom. The gold atoms were represented with “C5” beads. Parameters of the CG force field for the all-MUS AuNP can be retrieved from Salassi et al. [17]. In brief, the N1 and C1 beads of each MUS were connected with a harmonic potential with k_bond_ = 1250 kJ/ mol nm^2^ and with a harmonic cosine potential with k_angle_ = 25 kJ/mol nm^2^ and θ = 180 deg.

The Martini force field version 3 (PCT Souza, et al., Nat. Methods, 2021) [38] was applied to all proteins and to solvent using the *martinize.py* tool [39], and all simulations were performed with the Gromacs 5.0 package (Royal Institute of Technology and Uppsala University, Sweden. 2014) [40]. The all-MUS AuNPs were placed in the center of the simulation box, and one protein was randomly placed inside the box, as shown in Figure 9. The system was then solvated using the Martini representation.

For each protein type, three replicas were conducted by randomly changing the initial position of the protein and its velocities. The simulations were conducted in the NVT ensemble due to the rigid representation of the AuNP core that can lead to wrong pressure rescaling in the NPT ensemble. The timestep and the coupling time to the velocity rescale thermostat was of 10 fs, and the temperature was maintained at 320 K. Each simulation was performed for a total of 2 μs, for a total simulation time of 24 µs, which is only reachable using CG models.

## 5. Analysis

The binding site was defined by considering all backbone protein beads that were less than 4.5 Å from the all-MUS AuNP. This value was chosen based on the Martini representation, which is bigger than for atomistic simulations [28,41] but smaller than our previous CG models [9,11]. The calculation of the binding site was performed on the last frame of each simulation, when the binding of the protein was stable, in order to reduce the statistical fluctuations. The percentage of different types of amino acids (hydrophobic, hydrophilic, etc.) was retrieved from the total binding site for each protein.

The Root Mean Square Fluctuations (RMSF) were computed for both the free proteins and the proteins after binding. For the protein on the AuNP, we used the last 100 frames of each simulation where the binding was stable, i.e., when the Root Mean Square Deviation (RMSD) of the protein was almost constant.

The Potential of Mean Force (PMF) was obtained using the Umbrella sampling procedure. When the protein was stably attached to the AuNP, a force increasing in time was applied in the direction opposite to the one of the binding until the protein desorbed from the AuNP. The Weighted Histogram Algorithm Method (WHAM) was then applied to the configurations to obtain the PMF for each protein with all-MUS AuNPs [42,43].

The Solvent Accessible Surface Area (SASA) was calculated on the free protein and after binding. The probe radius was set to 0.21 nm, which is the radius of a water bead in the Martini model [38].

All analysis were performed using the tools in the Gromacs package [40].

## 6. Conclusions

We employed classical Molecular Dynamics to describe at the Coarse-Grained level the interactions of four common blood proteins with all-MUS AuNPs. We observed that the interactions were driven by hydrophobic amino acids with the MUS ligands that cover the AuNP surface. Moreover, these interactions were responsible of the strong binding of the proteins forming the corona around the AuNPs. We observed that binding with the all-MUS AuNPs did not change the secondary and tertiary structure of the proteins studied to an appreciable extent, suggesting that these all-MUS AuNPs have very small toxic effects. Finally, we derived a new method to compute the maximum number of proteins that can absorb over a AuNP based on Molecular Dynamic simulations that take into account the adaptation and conformational changes of proteins due to interactions with the capping ligands. The understanding of the interactions of AuNPs covered by ligands is still an important topic not only for the design of effective drug delivery techniques or nanomedicine applications, but also for the mitigation of side effect that these nanotechnologies can have on human health.

## Figures and Tables

**Table 1:** The contact probability for each amino acid in each protein and for the physico-chemical amino acid types. At the bottom of the table is the average value of the Potential of Mean Force (PMF) for each protein upon AuNP binding.

AA 1-Letter Code	AA Name	Hemoglobin	Serum Albumin	α1-Antiproteinase	Complement C3
A	Ala	13%	11%	7%	7%
C	Cys	2%	13%	0%	3%
D	Asp	2%	2%	4%	2%
E	Glu	0%	5%	9%	1%
F	Phe	10%	4%	11%	6%
G	Gly	2%	0%	0%	5%
H	His	3%	5%	0%	2%
I	Ile	0%	4%	9%	4%
K	Lys	8%	4%	4%	5%
L	Leu	3%	16%	20%	10%
M	Met	3%	2%	4%	4%
N	Asn	3%	4%	4%	2%
O	Pyl	0%	0%	0%	0%
P	Pro	10%	5%	2%	7%
Q	Gln	7%	4%	0%	7%
R	Arg	3%	2%	0%	5%
S	Ser	7%	2%	9%	9%
T	Thr	5%	2%	4%	6%
U	Sec	0%	0%	0%	0%
V	Val	15%	9%	7%	11%
W	Trp	2%	0%	2%	2%
Y	Tyr	2%	7%	4%	4%
Hydrophobic		48%	53%	64%	46%
Charged		14%	12%	17%	14%
Polar		22%	11%	17%	23%
Others		17%	24%	2%	17%
PMF (kcal/mol)		−41 ± 11	−36 ± 12	−36 ± 8	−47 ± 15

**Table 2:** Gyration radius in nm for the four proteins before (i.e., the free protein) and after binding with the all-MUS AuNP.

	Hemoglobin		Serum Albumin		α1-Antiproteinase		Complement C3	
Gyration Radius (nm)	before	after	before	after	before	after	before	after
	2.06 ± 0.02	2.06 ± 0.02	2.18 ± 0.03	2.23 ± 0.09	1.89 ± 0.01	1.90 ± 0.05	3.32 ± 0.02	3.29 ± 0.02

**Table 3:** In the first section, the mass, gyration radius, r_g_, and the smaller and bigger radius for each protein are recorded. In the second section, the maximum number of proteins that can absorb over the AuNP surface using different equations are reported.

Protein Size	Hemoglobin	Serum Albumin	α1-Antiproteinase	Complement C3
Mass (kDa)	64.74	133.14	44.38	185.69
R_g_ (nm)	1.47	2.64	2.16	4.59
Radius 1 (nm)	2.70	3.10	1.95	4.40
Radius 2 (nm)	2.80	3.65	3.00	6.65
N_max_				
Wang et al. [25]	7	2	3	1
	7	2	3	1
Calzolai et al. [10]	13	12	17	10
	13	11	12	8
Dell’Orco et al. [26]	12	11	16	8
	12	10	11	7
This work	5	5	6	3
